# Stephen Thomas Beale, M.D., D.D.S.

**Published:** 1900-02

**Authors:** 


### Obituary.

### STEPHEN THOMAS BEALE, M.D., D.D.S.

Dr. Stephen Thomas Beale, the oldest practitioner of dentistry in the city of Philadelphia, Pa., and one of the oldest graduates of the Jefferson Medical College, died at his home, Tulpehocken Street, Germantown, Tuesday evening, December 12, in his eighty-sixth year. His death was due to senile debility. He retained all his faculties up to the time of his death.

He was born in Brighton Sussex, England, May 23, 1814, and came with his parents to America in 1831, the family settling in Albany, N. Y.

He obtained his early education in England, and also attended the Albany Academy. Later he studied dentistry with Dr. McAllister of that place. In 1837 he came to Philadelphia, and attended lectures at the University of Pennsylvania and at Jefferson Medical College, taking up the study of both medicine and dentistry. He matriculated at the Jefferson College with Professor James Bryan as medical preceptor, and Dr. Lee as dental preceptor, and graduated from the college in 1847, receiving the degree of M.D.; and from that time until 1851 he practised both medicine and dentistry, starting in the latter profession in 1840.

The work of both professions, however, being too laborious, he devoted himself wholly to dentistry.

His dental practice covered a period of fifty-two years in Philadelphia, when he retired after a successful and lucrative practice.

He was known, with the late Dr. Ely Parry and Dr. John DeHaven White, as one of the "Fathers of Dentistry" in the city of Philadelphia, and, with them, was one of the founders of the Pennsylvania Association of Dental Surgeons, which was the first movement in that city for the promotion of scientific dental education.

He was the first vice-president upon its formation, and was the last survivor of its founders. Upon the fiftieth anniversary of the Association, Dr. James Truman, Dean of the Dental School of the University of Pennsylvania, who presided, referred to the period, fifty years ago, as one of transition in the history of dentistry, and the beginning of a new era in the study of science. He eulogized the founders of the organization, and said they could never have anticipated the number of societies and dental colleges which have been established since that period.

Dr. Beale was also instrumental in obtaining a charter for the Philadelphia College of Dental Surgeons, and on its formation was asked to fill two of its chairs, but was prevented from doing so by ill-health. The college, in 1853, conferred upon him the honorary degree of Doctor of Dental Surgery.

Dr. Beale was a master of his profession, and was interested in investigating and undertaking such cases as, in those days, were considered extreme,—as the repair of fractured maxilla and appliances for cleft-palate.

In his younger days his laboratory was fully furnished with all implements and utensils for the making of artificial teeth; carving entire and sectional blocks and single teeth; also for the smelting and refining of precious metals. For years this laboratory was thrown open to young students, and was well attended.

He was a man of broad views, advanced ideas, bright intellect, and great energy; an old-school gentleman of courteous manners, kindly heart, and domestic propensities; a thorough Latin scholar, a lover of nature and of the fine arts. He has contributed both to dental and literary magazines, and has published poems and other articles of merit, also many musical compositions. His active brain sought recreation in music, which he thoroughly understood and enjoyed.

He was a consistent, active member for thirty years with the Second Presbyterian Church of Germantown.

In his early life he married Miss Louise Boggs McCord, who died twelve years ago. They had a family of seven sons and three daughters, all of whom, with one exception, survive.

Two sons followed their father in the dental profession,—Dr. Stephen T. Beale, Jr., and Dr. Alonzo P. Beale, who died January 4, 1893; also three grandsons.

COMMUNICATED.

